# The effect of royal jelly and silver nanoparticles on liver and kidney inflammation

**Published:** 2021

**Authors:** Hossein Pourmobini, Mohammad Kazemi Arababadi, Mohammad Reza Salahshoor, Shiva Roshankhah, Mohammad Mohsen Taghavi, Zahra Taghipour, Ahmad Shabanizadeh

**Affiliations:** 1 *Immunology of Infectious Diseases Research Center, Research Institute of Basic Medical Sciences, Rafsanjan University of Medical Sciences, Rafsanjan, Iran*; 2 *Department of Immunology, Faculty of Medicine, Immunology of Infectious Diseases Research Center, Research Institute of Basic Medical Sciences, Rafsanjan University of Medical Sciences, Rafsanjan, Iran*; 3 *Department of Anatomical Sciences, Medical School, Kermanshah University of Medical Sciences, Kermanshah, Iran*; 4 *Department of Anatomy, Faculty of Medicine, Social Determinants of Health Research center Rafsanjan University of Medical Sciences, Rafsanjan, Iran*; 5 *Department of Anatomy, Faculty of Medicine, Rafsanjan University of Medical Sciences, Rafsanjan, Iran*; 6 *Department of Anatomy, Faculty of Medicine, Immunology of Infectious Diseases Research Center, Research Institute of Basic Medical Sciences, Rafsanjan University of Medical Sciences, Rafsanjan, Iran*

**Keywords:** Kidney, Liver, Nano-silver, Royal jelly

## Abstract

**Objective:**

Royal jelly (RJ) is a honey bee product for which, anti-inflammatory properties were shown *in vitro*. Nanoparticles, including nano-silver (NS), are plausible inflammation inducers that act by activation of immune cells and consequent production of pro-inflammatory cytokines. This project aimed to explore immunomodulatory effects of royal jelly and nano-silver on the kidney and liver.

**Materials and Methods:**

In this project, 40 male rats were grouped as follows: 10 rats as controls, 10 rats treated with RJ; 10 rats treated with both NS and RJ and 10 rats treated with NS. Liver and kidney interleukin (IL)-1β, -2, -6, and -33 levels were determined using commercial ELISA kits.

**Results:**

RJ reduced kidney IL-6 levels in comparison to control and NS--RJ groups. RJ and NS reduced kidney and liver IL-1β levels. Kidney IL-33 levels were decreased in the RJ and nano-silver groups in comparison to the NS--RJ group.

**Conclusion:**

Based on this study, it may be concluded that RJ together with NS can play anti-inflammatory roles and may affect the function of immune cells.

## Introduction

Royal jelly (RJ), as a viscous macromolecule, is produced by worker bees *(Apis mellifera)*, and is an essential food for queen of bees (Kamakura, 2011 ▶). Nowadays, RJ is widely used in production of several materials, such as dietary supplements and cosmetics (Ramadan and Al-Ghamdi, 2012 ▶). It has been reported that RJ has several bioactive properties, including anti-inflammatory (Ramadan and Al-Ghamdi, 2012 ▶), antiviral (El-Gayar et al., 2016 ▶), antibacterial (Nascimento et al., 2015 ▶), immunomodulatory (Pavel et al., 2011 ▶), and antioxidant (Delkhoshe-Kasmaie et al., 2014 ▶) activities. Thus, it was hypothesized that this product may be used as a protective material to decrease side effects of molecules like inflammation. Nano-particles are very small materials, which increase the effects of the drugs and chemical drugs increasing their interaction with their target molecules (Song and Kim, 2009 ▶). Nano-silver (NS) is a famous molecule that is used routinely with several drugs to increase their effectiveness (Sharma et al., 2009 ▶). However, it was documented that NS is associated with some side effects, including oxidation of DNA (Klingelfus et al., 2019 ▶), and cell cytotoxicity (Nakkala et al., 2017 ▶), and is harmful to the cardiovascular system, skin, gastrointestinal tract, respiratory tract and immune cells (Wang et al., 2016 ▶). Therefore, it was hypothesized that the use of some tissue protective materials, like RJ, with NS, may be useful to reduce the side effects of the nano-particle. Thus, this project was designed to explore the protective effects of RJ on the inflammatory properties of NS in rats. Due to the fact that the liver and kidney are two important tissues for detoxification, the kidney and liver levels of interleukin-1 (IL-1), -2, -33 and -6, as the pro-inflammatory cytokines, were determined in rats treated with NS, RJ and a combination of NS and RJ in this project.

## Materials and Methods

**Nano-silver particle and royal jelly**

In this project, the NS solution and RJ were obtained from Nanophishgaman Company, Mashhad, Iran and Pars Asal Company, Shiraz, Iran, respectively. The NS particles were at the concentration of 8000 ppm, less than 100 nm in size and of 99.98% purity, which was approved by the Company. RJ was prepared by solving of 20 g of RJ in 1 l of sterilized double-distilled water, as a previous investigation (Silici et al., 2009 ▶).

**Animals**

With respect to the ethics in animal experiments, monitoring nutritional status, standard light, and temperature and humidity conditions, 40 male Wistar rats, weighing 200-250 g, were divided into four groups randomly. Accordingly, 10 rats were considered controls, which did not receive any of the treatment materials; 10 rats were treated with 100 mg/kg RJ; 10 rats were treated with 30 mg/kg of NS and 100 mg/kg of RJ and 10 rats received 30 mg/kg NS. The NS particle solution, NS-RJ solution and RJ solution were daily prepared and given to the animals through gavage for 28 days. After 28 days of treatment with NS, NS-RJ, and RJ, the animals were killed by cervical amputation, under Ethical and standard conditions and their liver and kidneys were extracted under sterile conditions and transferred to the laboratory for cytokine assay. This project was granted by Rafsanjan University of Medical Sciences and approved by the related Ethical Committee (Ethical code: IR.RUMS.REC.1397.179).

**Cytokine assay**

The suspension of kidney and liver tissues were prepared in phosphate buffered saline (PBS) buffer and then, the kidney and liver levels of IL-1β, IL-2, IL-6 and IL-33 were measured using Karmania Pars Gene ELISA kits (Kerman, Iran) using the kits’ protocol.

**Statistical analysis**

The raw data were analyzed regarding their normal distribution and due to their normality, parametric tests were used to analyze them. Accordingly, the kidney and liver levels of IL-1β, IL-2, IL-6 and IL-33 among the groups were analyzed using One-Way ANOVA test by SPSS version 18. A p value less than 0.05 was considered significant. 

## Results

**Figure 1:**
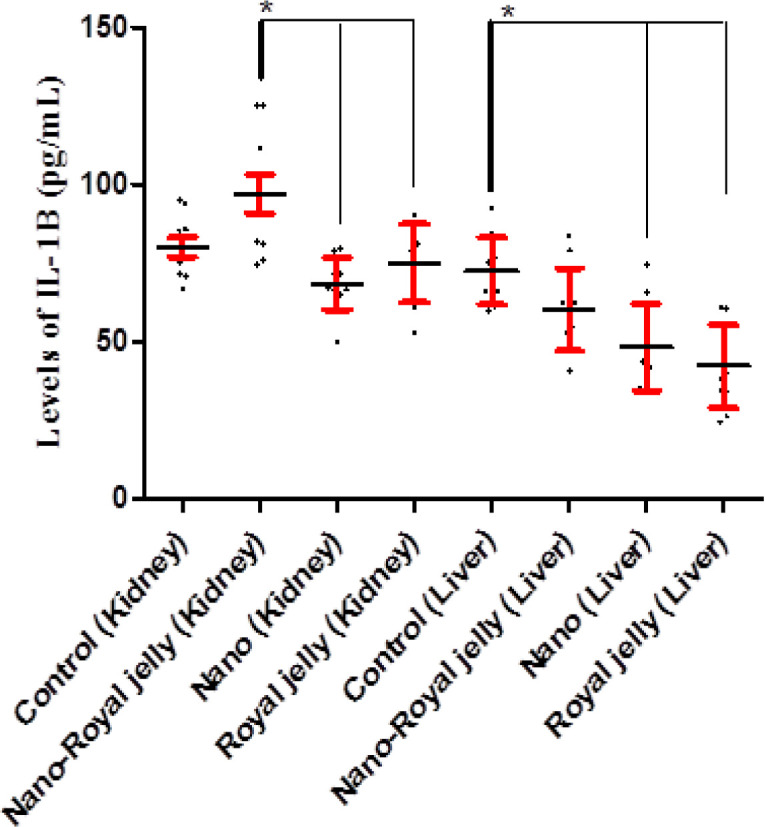
Kidney and liver levels of IL-1β in the control, and NS, NS-RJ, and RJ treated rats. The Figure shows that IL-1β was significantly higher in the kidney of the NS-RJ group compared to the NS (p=0.047) and RJ (p=0.002) groups and in the liver of the control group compared to the NS (p=0.025) and RJ (p=0.010) groups. *: p< 0.05

The results demonstrated that IL-1β was significantly higher in the kidney of the NS-RJ group than the NS (p=0.047) and RJ (p=0.002) groups and in the liver of the control group compared to the NS (p=0.025) and RJ (p=0.010) groups. Kidney levels of IL-1β in the controls were not different from other groups, including NS-RJ (p=0.266), NS (p=0.995) and RJ (p=0.639) groups (Figure 1). 

**Figure 2:**
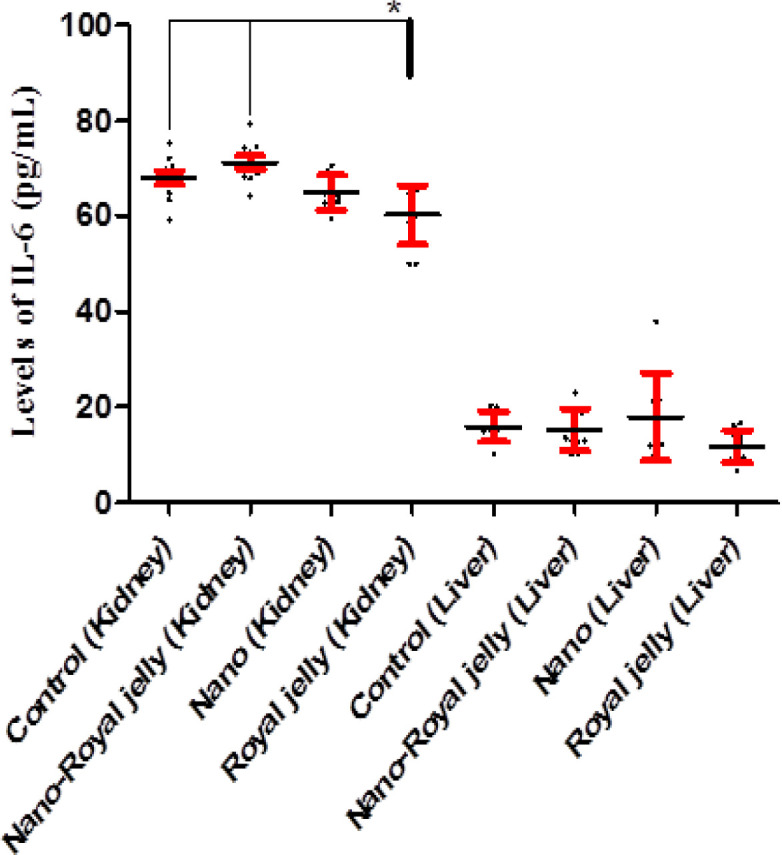
Kidney and liver levels of IL-2 in the control, and NS, NS-RJ, and RJ treated rats. Kidney (p>0.1) and liver (p>0.1) levels of IL-2 were not different among the groups

**Figure 3:**
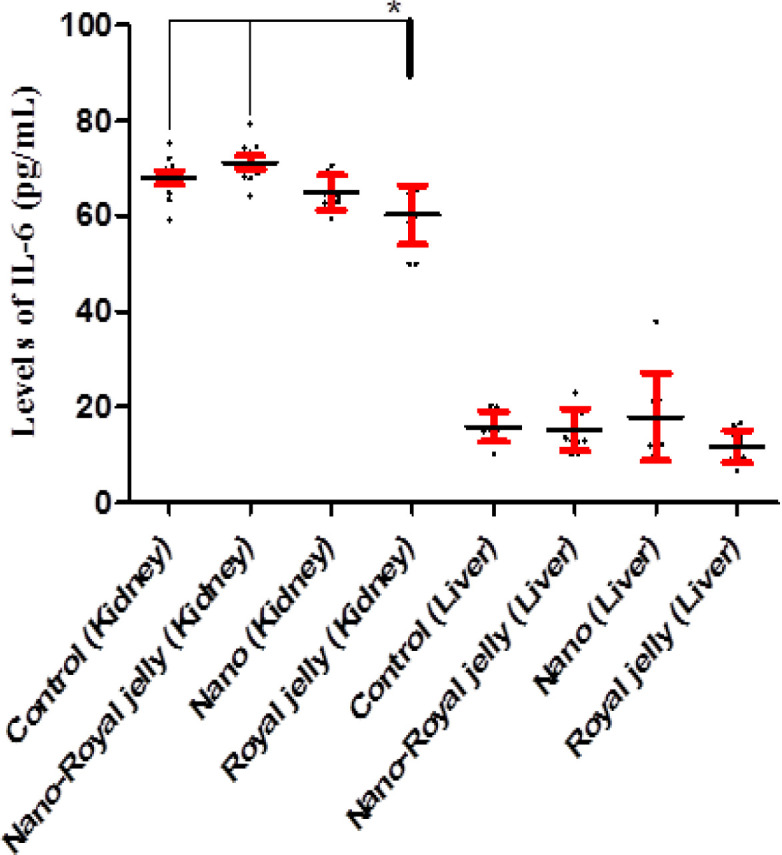
Kidney and liver levels of IL-6 in the control, and NS, NS-RJ, and RJ treated rats. Kidney levels of IL-6 were significantly higher in the control (p=0.046) and NS-RJ (p=0.002) groups compared to the RJ group. *: p<0.05

Kidney (p>0.1) and liver (p>0.1) levels of IL-2 were not different among the groups (Figure2). Kidney levels of IL-6 were significantly higher in the control (p=0.046) and NS-RJ (p=0.002) groups when compared to the RJ group. However, the liver levels of IL-6 did not vary among the groups (p>0.1) (Figure 3). 

**Figure 4:**
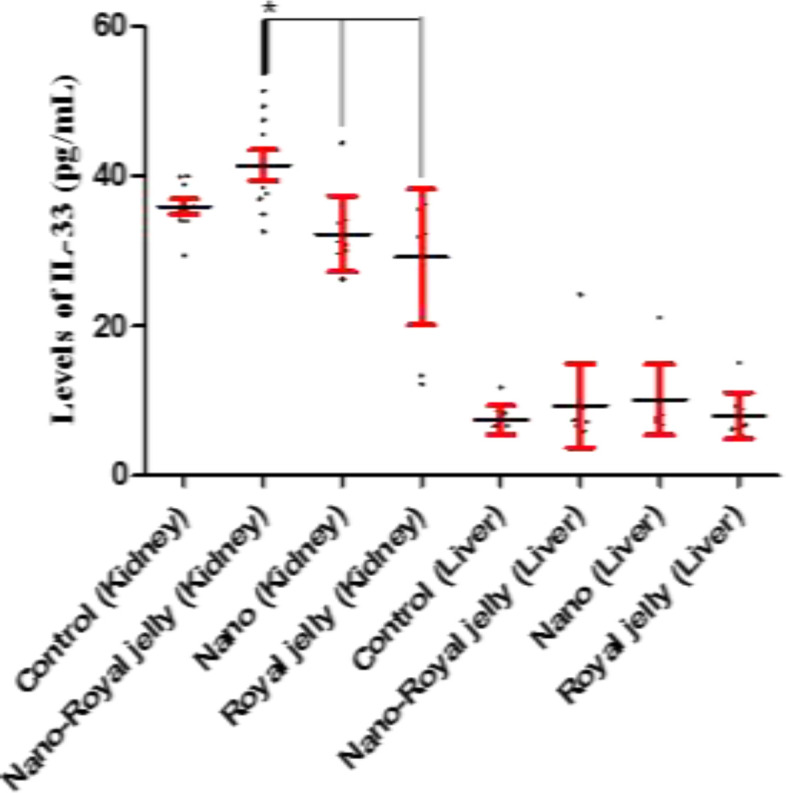
Kidney and liver levels of IL-33 in the control, and NS, NS-RJ, and RJ treated rats. Kidney levels of IL-33 were higher in the NS-RJ group compared to the NS (p=0.006) and RJ (p<0.001) groups

Kidney levels of IL-33 were higher in the NS-RJ group compared to the NS (p=0.006) and RJ (p<0.001) groups. But the liver levels of IL-33 did not vary among the groups (p>0.1) (Figure 4). As it is illustrated in Figures, kidney and liver levels of IL-1β, IL-2, IL-6 and IL-33 were significantly lower in the liver than kidney tissues (Table 1).

*: p<0.05; **: p<0.01; ***: p<0.001

## Discussion

The cytotoxic effects of NS were reported previously (Sharma et al., 2009 ▶). 

Therefore, it is plausible that NS suppresses the production of pro-inflammatory cytokines in the tissues, which are important to tissue repair and defense against microbes. Our results demonstrated that, although NS and NS-RJ were unable to alter the expression of IL-6 in comparison to the controls, RJ significantly decreased the cytokine in the kidney. Additionally, RJ significantly decreased kidney and liver levels of IL-1β and kidney levels of IL-33. Therefore, it appears that RJ has immunomodulatory effects in both the kidney and liver. IL-6 and IL-1β are important molecules that participate in the innate immunity-related pro-inflammatory diseases (Rahmanzadeh-Shahi et al., 2018 ▶; Borthwick, 2016 ▶). Additionally, the roles played by IL-33 in the pro-inflammatory disorders and deterioration of cancers were previously reported (Tong et al., 2016 ▶). Thus, it appears that RJ may be considered a future immunomodulatory agent. Although NS had anti-inflammatory effects in the current study probably due to its cytotoxicity leading to decreased functions of the immune cells, its combination with RJ neutralized immune-modulatory effects of RJ and resulted in up-regulation of IL-1β and IL-33 in the kidney and liver tissues, respectively. Due to the fact that IL-1β is the cytokine which needs to be activated by inflammasomes (Sepehri et al., 2017 ▶), while IL-6 is a pre-activated cytokine, it may be hypothesized that NS and RJ are effective on the inflammations. However, it needs to be explored by further investigations.

**Table 1:** Liver and kidney levels of IL-1β, IL-2, IL-6 and IL-33 among the evaluated groups

	Kidney							Liver	
Cytokine	Control	NS-RJ( (Nanosilver-Royal jelly)	NS (Nanosilver)	RJ (RoyalJelly)	Control	NS-RJ	NS		RJ
IL-1β	80.19±3.06	97.17± 6.11	74.75± 4.42	67.52± 7.08	72.44±3.36	60.29±4.16	48.37±4.34		46.20±6.27
IL-2	40.03±.59	37.37±3.77	38.82±1.04	34.55±2.21	14.08±.46	13.09±1.30	15.77±1.20		13.87±.57
IL-6	67.99±1.45	71.18±1.36	64.85±1.14	58.33±4.42	15.90±1.00	15.23±1.37	17.89±2.90		11.76±076
IL-33	35.88±1.01	41.38±2.05	32.22±1.60	29.10±2.87	7.39±.59	9.27±1.76	10.09±1.49		7.97±.97

Shin and colleagues showed that NS has potential cytotoxic effects on the peripheral blood mononuclear cells (PBMCs) and, hence are associated with decreased production of TNF-α by the cells (Shin et al., 2007 ▶). However, it has been demonstrated that high concentrations of NS are responsible factor to induce liver cell swelling and vacuolar degeneration (Nakkala et al., 2018 ▶). Chronic exposure to NS is also another reason for neuroinflammatory effects of NS (Locht et al., 2011 ▶). Therefore, it may be proposed that higher concentrations of NS and its chronic exposure to the tissues may have different results in rats which needs to explore by further investigations. Moreover, several investigators proved the protective roles played by RJ in the tissues, such as the testis (Ahmed et al., 2018 ▶), skeletal muscles (Metwally Ibrahim and Kosba, 2018 ▶), adipose tissue (Metwally Ibrahim and Kosba, 2018 ▶) and macrophages (You et al., 2018 ▶). Thus, it appears that the roles of RJ is dependent on other pro-inflammatory cytokines. As it is presented in Figure 1, the liver cytokine levels were not changed in the groups except IL-1β, which was decreased in the RJ and NS groups. Due to the fact that IL-2 is the main inducer of T lymphocyte clonal expansion (Au-Yeung et al., 2017 ▶), and considering to our results, it appears that neither NS-RJ component nor RJ or NS alone were able to affect adaptive immunity clonal expansion in both the kidney and liver.

According to this study, it may be imagined that RJ and NS together can play anti-inflammatory roles and may affect the function of immune cells. Therefore, using the RJ with NS may modulate NS suppressive effects on productions of key pro-inflammatory cytokines and may be considered for future studies. However, the function of RJ and its combination with NS on the immune cells needs to be explored by more investigations.
